# Evaluation of Incremental Validity of Casper in Predicting Program and National Licensure Performance of Undergraduate Nursing Students: Protocol for a Mixed Methods Study

**DOI:** 10.2196/48672

**Published:** 2023-10-18

**Authors:** Kathleen Stevens, Donna Moralejo, Renee Crossman

**Affiliations:** 1 Faculty of Nursing Memorial University St John's, NL Canada

**Keywords:** communication, empathy, incremental validity, mixed methods, nursing school admissions, problem-solving, professionalism, situational judgement testing, undergraduate nursing students

## Abstract

**Background:**

Academic success has been the primary criterion for admission to many nursing programs. However, academic success as an admission criterion may have limited predictive value for success in noncognitive skills. Adding situational judgment tests, such as Casper, to admissions procedures may be one strategy to strengthen decisions and address the limited predictive value of academic admission criteria. In 2021, admissions processes were modified to include Casper based on concerns identified with noncognitive skills.

**Objective:**

This study aims to (1) assess the incremental validity of Casper scores in predicting nursing student performance at years 1, 2, 3, and 4 and on the National Council Licensing Examination (NCLEX) performance; and (2) examine faculty members’ perceptions of student performance and influences related to communication, professionalism, empathy, and problem-solving.

**Methods:**

We will use a multistage evaluation mixed methods design with 5 phases. At the end of each year, students will complete questionnaires related to empathy and professionalism and have their performance assessed for communication and problem-solving in psychomotor laboratory sessions. The final phase will assess graduate performance on the NCLEX. Each phase also includes qualitative data collection (ie, focus groups with faculty members). The goal of the focus groups is to help explain the quantitative findings (explanatory phase) as well as inform data collection (eg, focus group questions) in the subsequent phase (exploratory sequence). All students enrolled in the first year of the nursing program in 2021 were asked to participate (n=290). Faculty will be asked to participate in the focus groups at the end of each year of the program. Hierarchical multiple regression will be conducted for each outcome of interest (eg, communication, professionalism, empathy, and problem-solving) to determine the extent to which scores on Casper with admission grades, compared to admission grades alone, predict nursing student performance at years 1-4 of the program and success on the national exam. Thematic analysis of focus group transcripts will be conducted using interpretive description. The quantitative and qualitative data will be integrated after each phase is complete and at the end of the study.

**Results:**

This study was funded in September 2021, and data collection began in March 2022. Year 1 data collection and analysis are complete. Year 2 data collection is complete, and data analysis is in progress.

**Conclusions:**

At the end of the study, we will provide the results of a comprehensive analysis to determine the extent to which the addition of scores on Casper compared to admission grades alone predicts nursing student performance at years 1-4 of the program and on the NCLEX exam.

**International Registered Report Identifier (IRRID):**

RR1-10.2196/48672

## Introduction

### Overview

There is an increased demand for registered nurses (RNs) with the increasingly complex health care system and aging population [1]. The strength of the global nursing workforce depends on the nursing education sector, and as a result, it is essential to invest in nursing education. Since the COVID-19 pandemic, there has been a 30% increase in applications to nursing programs [2]. Nursing education has limited resources to meet this demand [1], and there is increasing competition for seats [3]. The International Council of Nurses is concerned about this increased need for RNs and maintaining a high-quality workforce [2]. In their 2021 Strategic Workforce Plan for Nursing and Midwifery, the World Health Organization (WHO) stated that enough nurses need to be educated to have the “knowledge, competencies, and attitudes” to meet the population and health system’s needs [4]. Optimizing admission procedures and admitting applicants that are the best fit for the profession is a way to invest in nursing education, develop the nursing workforce, and support the WHO global strategic direction. This paper describes a research protocol for a multiphase mixed methods study. The purpose of the study is to assess the effectiveness of a situational judgement test, Casper, as part of the admission decisions in the Bachelor of Science in Nursing (BScN Collaborative) program in predicting student performance during the program and on the National Council Licensure Examination (NCLEX).

### Background

Admission criteria that accurately predict student success related to the performance of cognitive and noncognitive skills can help admit applicants with the prerequisites to develop nursing competencies. This can also help reduce the loss of invested resources from failure or attrition [3]. Cognitive skills refer to academic achievement and “involve the ability to understand complex ideas, to adapt effectively to the environment, to learn from experience, to engage in various forms of reasoning, to overcome obstacles by taking thought” [5]. Noncognitive skills comprise “personal traits, attitudes, and motivations” [6]. It is important to note that noncognitive skills usually involve mental processes such as decision-making, interpersonal skills, and emotional maturity, but they are more difficult to measure than cognitive skills (Textbox 1) [7].

**Description of key terms. Casper measures collaboration, communication, empathy, equity, ethics, motivation, problem solving, professionalism, resilience, and self-awareness.**

**Cognitive skills**

- Academic achievements
- Ability to understand complex ideas, learn from experience, and reason [5]

**Noncognitive skills**

- Personal traits, attitudes, and motivation (eg, empathy) [6]
- Involve mental processes (eg, decision-making and interpersonal skills)

**Situational judgment tests (SJTs)**

- A methodology for assessing decisions in relation to underlying knowledge, skill, traits, and other attributes [8]

**Casper**

- A web-based SJT (90-110 minutes) that assesses noncognitive skills
- Test-takers respond to scenarios concerning what they would do and why
- Trained raters score the test
- Interrater reliability and intraclass correlations range from 0.84 to 0.94 [9]

Applicants who have appropriate decision-making skills that reflect capabilities related to noncognitive skills may be more suitable for the nursing profession. Noncognitive skills related to areas such as communication, empathy, problem-solving, and professionalism are essential to assume the roles of an RN (eg, advocate, coordinator of care, and educator) and deliver competent care to often complex patients and families across the life span in different settings (eg, acute care and the community).

There is strong evidence that cognitive ability predicts program success, but there is limited evidence related to noncognitive skills. The authors of a 2020 systematic review of 16 cohort studies explored preadmission criteria that predicted student success in undergraduate nursing programs in the United States. These authors reported that it was well established that cognitive predictors such as admission grade point average (GPA) predicted program success. Although not used as admission criteria, age, gender, and ethnicity also predicted success. Other possible predictors included grades on standardized tests, such as the Test of Essential Academic Skills. It is important to note that the definition of success used by most studies varied and was related to academic success, passing the licensure exam, attrition, program completion, and GPA in nursing and science courses. These authors contend that it is difficult to isolate a single variable as the best predictor of student performance, and it would be more reliable to use a combination of variables [1]. No research has considered the predictive validity of noncognitive skills on student performance as well as the value of adding these criteria, that is, the incremental validity, to the nursing admission process. Incremental validity is defined as “the improvement obtained by adding a particular procedure or technique to an existing combination of assessment methods. In other words, incremental validity reflects the value of each measure or piece of information to the process and outcome of assessment” [10].

One possible approach for nursing admissions is using situational judgment tests (SJTs) to measure noncognitive skills, combining these results with cognitive criteria. SJTs are defined as “a methodology for assessing how an individual’s underlying knowledge, skills, traits, and other attributes are expressed when making decisions about job-related scenarios” [8]. The use of SJTs has been identified as a valid approach for selecting medical school applicants concerning the performance of nonacademic skills. The authors of a systematic review of 30 studies (10 observational, 17 cohort with follow-up from 1-9 years, and 3 observational with longer follow-up) conducted a meta-analysis of 26 of these studies that reported validity correlations related to SJT and student performance. A pooled correlation was obtained of 0.32 (95% CI 0.26-0.39; *P*<.001) [11]. A total of 15 of the 17 studies that assessed the incremental validity of adding SJT found modest values of 5%-10% of additional variance accounted for (ie, the *R*^2^ of regression models with and without SJTs differed by 5-10 percentage points). This suggests that there is value to adding SJTs, but SJTs alone cannot account for the outcomes assessed. The authors point out that this is a modest relationship but was considered in the context of medical school admissions, where many applicants are strong. Therefore, detecting slight differences is difficult [11].

The authors of a 2020 scoping review reported that no research was identified between 2006 and 2019 concerning using SJT for nursing school admissions [12]. A literature search did not identify any research published after 2019. Research is needed to explore the predictive nature of SJTs to inform nursing admissions decisions. Nursing admission committees need evidence of the incremental validity of admission criteria that can predict student success both academically and in noncognitive skills [13].

The BScN (Collaborative) program at Memorial University of Newfoundland (MUN) is a competitive program composed of 2 admission options: the 4-year option and the accelerated option. Over 600 applicants per year apply for 291 seats at 3 sites. Historically, admission decisions were primarily based on academic performance, but starting in 2021, the admissions process was modified to include Casper, a SJT that assesses noncognitive skills. The Casper score, the applicants’ academic average, and references were used in the admissions decisions.

Casper is a web-based test developed by Acuity Insights that takes 90-110 minutes to complete. Test-takers are asked to respond to various scenarios that are designed to measure noncognitive skills concerning what they would do and why. The goal of these scenarios is to ascertain “behavioral tendencies” of individuals who are applying to programs that are person-centered. The test is divided into 2 sections: a video response section and a written response section. Trained raters score the test, and within 2-3 weeks of completion, the Casper score, converted to a *z* score, is sent to the program the applicant applied to. Casper assesses for the following noncognitive skills: collaboration, communication, empathy, equity, ethics, motivation, problem-solving, professionalism, resilience, and self-awareness. As noted above, these noncognitive skills are essential for nursing practice. The interrater reliability of Casper is strong, with intraclass correlations ranging from 0.84 to 0.94 across 55,988 applicants (Textbox 1) [9].

The goal of using Casper in our nursing program was to guide the admission of applicants with the best potential for success in the program and the profession. The need to include the assessment of noncognitive skills during the admissions process was further reinforced by findings of the 2016-2018 Employer Survey Report (prepared by BScN Collaborative Program), which raised concerns about recent graduates’ performance in the clinical area with professionalism, empathy, communication, and problem-solving. These concerns were also highlighted in discussion with the College of Registered Nurses of Newfoundland and Labrador, the admissions committee, and faculty members.

This study proposes a mixed methods research (MMR) study to address and add to the knowledge related to predictors of success in a nursing program concerning noncognitive skills and program success. In particular, the predictive nature of Casper will be examined concerning optimizing admission decisions and admitting applicants with the greatest potential of completing the program, meeting the competencies of the nursing profession, and passing the NCLEX.

### Purpose

The purpose of this multiphase MMR study is to assess the effectiveness of Casper as part of the admission decisions in the BScN (Collaborative) program in predicting student performance in years 1, 2, 3, and 4 of the program, and on the NCLEX, compared to academic performance alone. Overall academic success will be evaluated, as well as performance in psychomotor skills laboratory sessions and clinical settings. Problem-solving, empathy, and other skills are expectations in both types of settings, and all are expected to improve as students progress through the program.

The overarching MMR research question is, What is the effectiveness of the addition of Casper to admission decisions in predicting student success across the curriculum? The objectives of the quantitative and qualitative analysis and the integration of both are as follows:

1. To assess the incremental validity of Casper scores in predicting student performance with communication and problem-solving and student behavior related to professionalism and empathy at years 1, 2, 3, and 4 of the program, and on NCLEX performance, compared to academic ranking alone.
2. To examine clinical and laboratory faculty members’ perceptions of student performance, challenges, and influences related to professionalism, empathy, communication, and problem-solving in psychomotor skills laboratory sessions and the clinical area.

## Methods

### Ethical Considerations

We obtained ethical approval (20222324) from the Newfoundland and Labrador Health Research Ethics Authority in February 2022, and this approval was renewed in January 2023.

### Study Design and Setting

**Figure 1:**
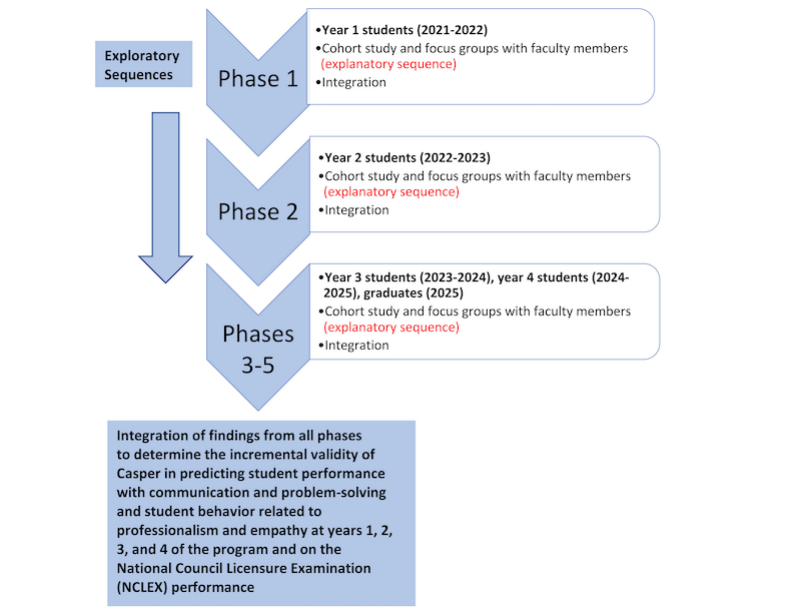
Mixed methods model that illustrates the exploratory and explanatory sequences and points of integration.

This study will use a multistage evaluation MMR study design with 5 phases and 2 embedded sequences (Figure 1). An interpretive description was used to guide the qualitative portion of the study. Both MMR and interpretive description are underpinned by pragmatism, and as such, both objective and subjective knowledge are valued and used to answer the research questions [14,15].

In each study phase, there is an embedded explanatory sequence whereby quantitative data collection (longitudinal cohort study) will be followed by qualitative data collection (focus groups). The goal of the qualitative interviews is to help explain the quantitative findings and gain a more robust understanding of student performance concerning professionalism, empathy, communication, and problem-solving. The other embedded sequence is an exploratory sequence, whereby what is learned in each phase will inform focus group questions in the subsequent phase.

### Student Recruitment

In March 2022, all first-year students in the 4-year option and accelerated option at all 3 sites were invited to participate. These students were admitted in 2021 and were the first cohort to complete Casper as part of the admission process. Students who chose to participate will be followed through their program to the completion of the NCLEX exam. A faculty member at each site not associated with the study acted as an intermediary and introduced the study using a script. If students were interested, an investigator came to the class to further explain the study, and an information letter was provided to students. At that time, informed consent was obtained from students wanting to enroll, and the first set of questionnaires was completed. Students who chose not to enroll at that time were told that if they decided they would like to enroll, they could contact the research assistant within 1 week. It was explained to students in the consent form that participation (or not) would not affect any courses that they take in the nursing program; there were no known risks to participating in the study; all identifying information would be kept confidential; study data would be anonymized and safely stored using a secured drive; and only the research team would have access to the data.

A demographic profile was completed for each student (eg, age, gender, Canadian province or country of origin, and previous education). The student’s Casper score and admission GPA for those who had completed university courses, or the high school average, were obtained through the Nursing Admissions Office. To access these data, a conditional agreement was reached between the university and the researchers.

### Faculty Recruitment

Focus groups with clinical and laboratory faculty will be conducted at the end of each year of the program at a mutually convenient location and time. Recruitment is done by email using purposive convenience sampling. Emails will be sent to clinical and laboratory faculty that taught this group of students in the previous year. During the focus groups, we explore faculty perceptions of student performance, challenges, and influencing factors related to noncognitive skills in the laboratory and clinical settings. Written informed consent is obtained from participants in the focus groups. It is explained in the consent form that all information that identifies participants will be kept confidential, and only the research team has access to the data. As well, it is explained that there may be potential risks, including being uncomfortable sharing experiences in the context with others in the focus group. Every effort is made by the researchers to ensure participant comfort as well as safety during the focus group (eg, the researchers will ask the participants to respect the privacy of fellow participants and treat all information shared with the group as confidential).

### Sample Size

To assess incremental validity, the *R*^2^ of regression models, with and without SJTs, are compared to see if the addition of the SJT explains more of the variance found. The sample size of this study was calculated using the findings of a previous study [16] that assessed incremental validity using *R*^2^ analysis. In it, researchers examined the incremental validity of adding SJTs to the use of a clinical problem-solving tool (CPST) for predicting performance in workplace simulation exercises as part of the selection process for postgraduate medical training. The researchers found that a regression model with CPST but not SJT explained 35% of variance (*R*^2^=.35) whereas a model with both CPST and SJT explained 57% of the variance (*R*^2^=.57) seen in the simulation scores. Their sample size of 135 was sufficient to detect this difference. Our target sample size is, therefore, 135, assuming we will see similar differences for the addition of SJT to admission grades (instead of CPST) on different outcomes. Few of the other studies reported in the systematic review by Webster et al [11] assessed outcomes similar to ours, few related to admission to medical schools, and most were conducted on very large (pooled) cohorts of medical students (N>1000) making it difficult to identify the minimum sample size required. No data are available related to nursing students. Assuming an attrition rate of 10%, which is normally seen in our program, 150 students will be recruited.

### Quantitative Data Collection

**Figure 2:**
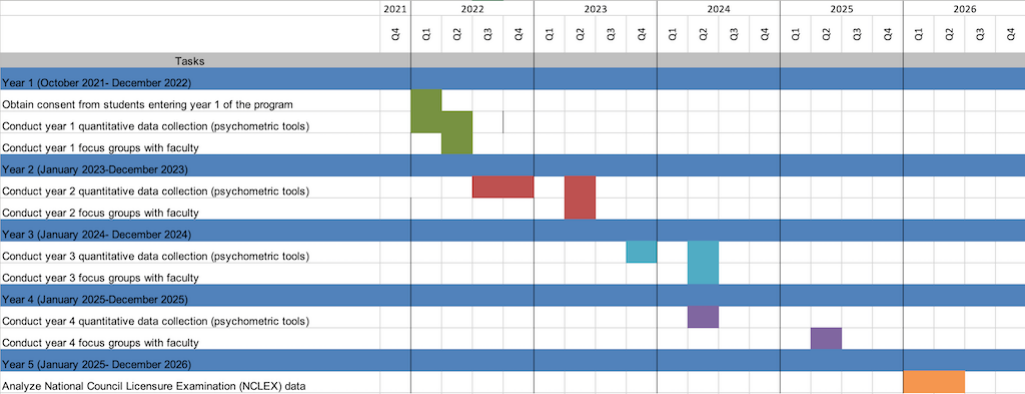
Timeline for data collection (2022-2026).

This study has 5 phases, with quantitative data collection occurring in appropriate psychomotor skills courses during each academic year and at the end of each phase (ie, end of each year and after NCLEX completion). Figure 2 depicts the timeline for data collection.

### Outcomes and Measures

The following outcomes, identified as concerns related to graduate performance and entry-level competencies of RNs, will measure nursing student performance: communication, problem-solving, empathy, professionalism, GPA and grades, and NCLEX success.

Communication is being evaluated during psychomotor testing in a nursing skills laboratory session using a tool developed for the study. One specific course was chosen for each year of the program. In these courses, students learn and practice foundational skills (eg, health assessment, basic care, and safe medication administration) and complex psychomotor skills (eg, complex dressings, care of chest tubes, and care of central lines). Nursing skills laboratory session testing is scenario-based and requires students to demonstrate effective communication skills (eg, verbal skills and documentation). Laboratory faculty who evaluate students during the psychomotor testing, use consistent rubrics for that course, and complete these tools based on the student’s performance related to communication. The communication tool assesses whether students introduce themselves, use appropriate language, and complete appropriate documentation. There are additional criteria to assess if the testing involves a real person (eg, the student used appropriate listening skills). The possible range of scores for this assessment tool is 0-14, with higher scores indicating strong communication skills. Face validity was established by having faculty who have extensive experience conducting psychomotor assessments with nursing students review the tool to ensure the content was appropriate and relevant. The interrater reliability score was 100%. Laboratory faculty received training on how to complete the tool (Multimedia Appendix 1).

Problem-solving is being evaluated during psychomotor testing in a nursing skills laboratory session using a tool developed for the study. One specific course has been chosen for each year of the program. Nursing skills laboratory session testing is scenario-based and requires students to demonstrate effective problem-solving skills. Laboratory faculty who evaluate students during the psychomotor testing, using consistent rubrics for that course, will complete this tool based on the student’s performance related to problem-solving. The problem-solving tool evaluates whether the student recognizes the problem, attempts to address the problem, and takes appropriate action to address the problem. The possible range of scores for this assessment tool is 0-6, with a higher score indicating strong problem-solving skills. Face validity was established by having faculty who have extensive experience conducting psychomotor assessments with nursing students review the tool to ensure the content was appropriate and relevant. The interrater reliability score was 94%. Laboratory faculty received training on how to complete the tool (Multimedia Appendix 2).

Empathy will be measured with the Jefferson Scale of Empathy (reliable and valid). This 20-item self-report scale takes approximately 10 minutes to complete and measures 3 constructs: “perspective taking,” “compassionate care,” and “standing in a patient’s shoes.” Each item is answered using a 7-item Likert scale (1=strongly agree and 7=strongly disagree). The scores can range from 20 to 140, with higher scores indicating stronger empathy [17].

Professionalism will be measured with an adapted Nurses Professional Values Scale-Three (NPVS-3; reliable and valid). This measurement is a 28-item self-report scale. The demographics questions were removed as the questions were either not appropriate for our context or the information would be obtained from the MUN Registrar’s Office. Professional nursing values are evaluated using a 5-item Likert scale: not important (A), somewhat important (B), important (C), very important (D), and most important (E). Numeric values were assigned to each letter and added together to get the total score. The scores can range from 28 to 140. A higher score indicates a stronger nurse’s professional value orientation [18].

For GPA and grades, GPA (yearly and cumulative) and marks in academic nursing courses will be obtained at the end of the academic year from the registrar’s office.

For NCLEX success, data will be obtained from the College of Registered Nurses of Newfoundland and Labrador (CRNNL) regarding NCLEX success in terms of pass or fail. If available, as NCLEX scoring is changing, the overall performance (logit score) and performance on individual test categories (logit score) will be obtained. The CRNNL is the regulatory body and professional association for all RNs and nurse practitioners and the custodian for the National Council of State Boards of Nursing data. An agreement was signed between the researchers, MUN, and CRNNL to access the data.

### Predictor Variables

Data will be collected on several predictor variables. Admission grades and Casper scores are the predictor variables of interest (ie, the independent variables to be assessed in terms of predictive and incremental validity). The other variables will be assessed and controlled for as potential confounders.

- Admission grades: the admission GPA will be obtained for students who had some postsecondary education before admission; the admission high school average will be obtained for students admitted directly from high school. These data will be obtained from the registrar’s office.
- Admission Casper score: students complete Casper as part of their admissions application. Results will be obtained from the nursing admission office.
- Demographics: age, gender, province, and program of study (ie, 4-year option and accelerated option) and program site, as the BScN (Collaborative) program is offered at 3 sites. These data will be obtained from the nursing admissions office.

### Quantitative Data Collection Procedures

At the end of each year of the program, the research assistant or researchers will meet with the students to complete the 2 pen and paper questionnaires: the Jefferson Scale of Empathy and the NPVS-3. The research assistant has been trained in data collection and the use of measurement tools. Students are monitored to ensure they are not talking to each other. As a thank-you for participating, students are given a CAD $10 (US $7.32) coffee card at each phase of data collection. The completed problem-solving and communication tools will be obtained directly from the laboratory instructors.

Completed questionnaires and tools at the sites are placed in a sealed envelope and sent to the MUN Faculty of Nursing Research Unit by courier. If there is a delay in sending the completed questionnaires by courier, the questionnaires are stored in a locked filing cabinet in one of the researcher’s offices. Data will be obtained from the Registrar’s Office, Nursing Admissions Office, and CRNNL at the appropriate times, using procedures established with each source.

### Qualitative Data Collection

Qualitative data collection will occur at the end of each academic year. Figure 2 depicts the timeline for data collection. Both primary investigators (PIs) have expertise in qualitative research and experience in focus group facilitation. The PIs will conduct the focus groups at the MUN Faculty of Nursing and will provide instruction, guidance, and support for focus group facilitators at the other 2 sites. Focus group interviews with clinical and laboratory faculty will be recorded and transcribed verbatim. An interview guide is used (Textbox 2). Interpretive description will be used to guide data collection, and consistent with this approach, initial focus group questions were guided by the research purpose and objectives [17]. Subsequent interview guides will be developed based on the unfolding data analysis [19,20]. With interpretive description, the researcher goes beyond description and asks what it means, what I can do with these findings, and why it is important [15]. Quirkos (version 2.5.2; Quirkos), a qualitative software package, will be used to categorize the data to identify themes.

**Phase 1 faculty focus group questions.**

**Faculty focus group questions**

1. Tell me about your experiences with students this past year in the clinical setting concerning communication, professionalism, empathy, and problem-solving.
2. Tell me about your experiences with students this past year in the laboratory setting concerning communication, professionalism, empathy, and problem-solving.
3. Did you experience any challenges related to communication, professionalism, empathy, and problem-solving?
4. What do you think influenced their performance in relation to communication, professionalism, empathy, and problem-solving?

### Statistical and Analytic Plan

Descriptive statistics (eg, means and proportions), as appropriate for the data, will be used to describe the characteristics of the participants, including their GPAs and their scores on the different outcome measures. Stata (version 17; StataCorp) will be used to analyze the data. The data will be checked for the amount of missing data; if they are missing completely at random, and if warranted, multiple imputation will be used. Different analyses will be conducted to address each research objective, as described below.

For objective 1 (phases 1-5), hierarchical multiple linear regression will be conducted to determine if Casper is a significant predictor of each outcome (predictive validity) and if a model with both Casper and admission grade explains more of the variance for the outcome than a model without Casper (incremental validity). Each model assessed will contain both the Casper score and the admission average and include potential predictors and confounders (eg, GPA for the year, age, gender, province, program of study, and program site). Modeling will be conducted separately for students who entered directly from high school (admission grade=high school average) and those who had some postsecondary education before admission (admission grade=GPA for the postsecondary courses). The modeling will be repeated at the end of each year (years 1-4) for each of the yearly outcomes (ie, dependent variables): communication, problem-solving, empathy, professionalism, and GPA. The measures used will be the scores for that year.

For the first model that is tested, all the predictor variables will be entered into the model. The predictor variable with the highest *P* value (*P*>.05) will be dropped, and the model without this variable will be tested. The second model will be compared to the previous model using the likelihood ratio (LR) test. If the LR test is not significant (*P*>.05), the variable is not a predictor and needs to be assessed for confounding before being removed from the model. To assess confounding, the coefficient for the main exposure of interest is assessed to see if it changes when the potential confounder is taken out of the model. If it did, that variable would be considered a confounding variable and would be kept in the model. If the coefficient did not change, the variable is not a confounder and will be removed from the model. This process will be repeated, comparing it with the previous model, until all of the variables are tested and the final model has been established. To determine the incremental validity, the *R*^2^ (ie, the percentage of variance explained by the model) for the final model and the *R*^2^ for that model without the Casper score will be compared.

A similar analysis will be conducted in 2025 using logistic regression to determine if Casper is a significant predictor of NCLEX success (predictive validity) and if a model with both Casper and admission grade explains more of the variance for NCLEX success than a model without Casper (incremental validity).

For objective 2 (phases 1-5), under the direction of the methodological approach of interpretive description, the analytical framework, the three Cs (coding, categorizing, and concepts) will be used to conduct a thematic analysis of the data. The following 6 steps are used in this iterative process: initial coding, revisiting initial coding, developing categories, revising the initial list of categories based on additional review of the data, reviewing categories and subcategories, and developing themes (ie, concepts) [21]. The PIs will independently code the focus group data and reach consensus on the themes [19]. We will use verification strategies to incrementally build rigor throughout the qualitative phase of the project [19,22]. These strategies include methodological coherence (eg, ensuring the fit between methods of data collection and analysis with the research design) and concurrently collecting and analyzing data (eg, ensuring that future focus group questions are based on the analysis and findings of previous focus groups) [19,22]. Both PIs and other focus group facilitators are known to faculty participants. Another verification strategy, researcher responsiveness, offers the opportunity for the PIs to reflexively consider their influence on the research [19,22]. For example, debriefing after each focus group to explore how we questioned, probed, and, overall, facilitated the group to meet the goals of the research. These verification strategies will enhance the trustworthiness and credibility of the data [19,22,23].

The integration will occur after each phase is complete and at the end of the study.

- Explanatory sequences (end of phases 1-5): integration with this design aims to use the qualitative results to explain the quantitative data [14]. Each data set will be analyzed separately (ie, questionnaires completed by students, problem-solving and communication tools completed by faculty, and focus groups with faculty), and then the “fit of the data integration” will be analyzed. This refers to the coherence of the qualitative and quantitative findings; completing this analysis will result in a more comprehensive understanding of the findings. Three possible outcomes will be assessed. (1) Confirmation occurs when the findings from both data sets confirm the results of the other: the 2 data sources provide similar conclusions, and therefore, the results have more credibility. (2) Expansion occurs when the findings from the 2 data sets diverge and expand insights related to the findings. And (3) discordance occurs if the findings of qualitative and quantitative data sets are inconsistent or disagree [24].
- Exploratory sequences (end of phases 1-3): the goal of integration in an exploratory sequence is to build from one database to another [14]. In this study, what is learned in each phase will inform qualitative data collection for the focus group in the subsequent phase.

## Results

This study was funded in September 2021. In March 2022, a total of 144 first-year students enrolled in the research, who will be followed throughout the nursing program to the completion of the NCLEX exam in 2026. A total of 3 focus groups with clinical and laboratory faculty were conducted in May 2022. Data analysis of year 1 data is complete, and data analysis of year 2 data has begun. STROBE (Strengthening the Reporting of Observational Studies in Epidemiology) guidelines for cross-sectional research and COREQ (Consolidated Criteria for Reporting Qualitative Research) guidelines for qualitative research will be followed when results are presented [25,26]. The knowledge translation includes reports, presentations, and publications. Year 1 and a portion of year 2 data were presented at the International Council of Nurses Congress in July 2023.

## Discussion

The results of this study may provide evidence to support the use of the SJT, Casper, to guide nursing admissions to optimize the selection of applicants who have the greatest potential of completing the nursing program and meeting the competencies of the nursing profession. Specifically, the results will show if Casper helps predict the performance of the 4 noncognitive skills that were measured (ie, communication, problem-solving, empathy, and professionalism) and success on the NCLEX. Identifying and selecting applicants who perform well in these areas will mean that individuals are selected who are a good fit for nursing and therefore may be more likely to remain in the program and be licensed to practice as an RN. This will, in turn, potentially improve the quality of the nursing workforce and patient care.

These findings will have implications for nursing education and administration concerning admissions procedures and whether the use, or continued use, of SJTs is warranted based on the available evidence. As well, the results of this study will hold implications for nurse researchers to further evaluate the use of SJTs such as CASPER. Consideration should be given to conducting a multisite study across several university schools of nursing and comparing student cohorts on outcomes measured by Casper (eg, communication and problem-solving) before and after the implementation of Casper.

There are several notable strengths. This is the first study to report on the predictive ability of situational judgment testing as part of nursing admission criteria. The recruitment of students at 3 nursing schools ensured that participants were representative of the target population. Also, using a multiphase MMR approach and integrating or merging quantitative and qualitative data allows for a more comprehensive answer to the research questions [14]. Concerning this study, using MMR provides a greater understanding across the years of student performance. It also increased understanding of the challenges and influences related to professionalism, empathy, communication, and problem-solving. Using focus groups as the data collection method in the qualitative phase allows for the development of rich, thick descriptions and, thus, ultimately, enhanced understanding of the concepts explored [27]. Finally, using regression in the analysis allows for control of confounding variables, compared to just correlations between Casper and admission grades with the outcomes.

There may be some possible limitations to this study. The first is the attrition of participants throughout the study. We anticipate that there will be some attrition from the study with student failure and withdrawal from the study or program throughout the 5-year study timeline. The COVID-19 pandemic has greatly influenced this cohort, and this may be found to be a contributing factor to attrition and student performance. The second limitation concerns variables that are measured with self-report (eg, professionalism and empathy). Although the tools used are valid and reliable, there is a potential for social desirability bias with these measures. Additionally, while focus groups for data collection are considered a strength, there is the possibility of power imbalances within the group, thus limiting the breadth and depth of the data [27,28]. This will be mitigated by facilitators, who will establish guidelines at the beginning of the focus group and provide opportunities for all participants to participate in the discussion, including the exploration of new ideas as they emerge. The final limitation concerns potential differences in the student experience across the 3 program sites. Although the same curriculum is offered to students at the 3 program sites, there may be some variability in school culture, clinical experiences, and curriculum delivery that could impact the outcomes that are measured. The program site will be controlled for in the regression analysis. These potential limitations will be taken into consideration when interpreting the results.

The addition of a SJT such as Casper may be a strategy that could support admissions committees as they endeavor to admit students that have both the cognitive and noncognitive skills to be successful in a nursing program. SJTs have been widely used for medical education, but there is limited literature on their usefulness for nursing programs. This multistage evaluation mixed methods study will add to the literature about the predictive ability and incremental validity of the SJT Casper. The findings of this study will have implications for nursing education and research.

## Appendix

Multimedia Appendix 1 Communication tool.

Multimedia Appendix 2 Problem Solving Tool.

## Abbreviations

BScN: Bachelor of Science in Nursing
COREQ: Consolidated Criteria for Reporting Qualitative Research
CPST: clinical problem-solving tool
CRNNL: College of Registered Nurses of Newfoundland and Labrador
GPA: grade point average
LR: likelihood ratio
MMR: mixed methods research
MUN: Memorial University of Newfoundland
NCLEX: National Council Licensing Examination
NPVS-3: Nurses Professional Values Scale-Three
PI: primary investigator
RN: registered nurse
SJT: situational judgment test
STROBE: Strengthening the Reporting of Observational Studies in Epidemiology
WHO: World Health Organization

## Data Availability

The data sets generated and/or analyzed during this study are not publicly available due to participant confidentiality but are available from the corresponding author on reasonable request.
