# Supplementary material for: Evaluation of Incremental Validity of Casper in Predicting Program and National Licensure Performance of Undergraduate Nursing Students: Protocol for a Mixed Methods Study
Source: JMIR Res Protoc. 2023 Oct 18;12:e48672. doi: 10.2196/48672 (PMC10620628; doi:10.2196/48672)
Supplement: Multimedia Appendix 1 [file resprot_v12i1e48672_app1.docx]

**Assessment Tool: Communication**

**Part A: To be used in all psychomotor testing.**

| **Item** | **Yes, without prompting (2 points)** | **Yes, with prompting**  **(1 point)** | **No**  **(0 points)** | **Comments** |
| --- | --- | --- | --- | --- |
| 1. **The student communicated using appropriate language.** |  |  |  |  |
| 1. **The student**   **introduced self** |  |  |  |  |
| 1. **The student completed appropriate documentation** |  |  |  |  |

**Part B: To be used in addition to Part A if the psychomotor evaluation involves a real person.**

| **Item** | **Yes, without prompting**  **(2 points)** | **Yes, with prompting**  **(1 point)** | **No**  **(0 points)** | **Comments** |
| --- | --- | --- | --- | --- |
| 1. **The student used non-judgmental language and tone** |  |  |  |  |
| 1. **The student used appropriate listening skills** |  |  |  |  |
| 1. **The student used appropriate responses** |  |  |  |  |
| 1. **The student evaluated the person’s understanding of the communication.** |  |  |  |  |

**Directions and Scoring:**

This assessment will be completed by faculty evaluating students during psychomotor skills testing. Faculty will be provided with education regarding how to use this assessment tool.

**Part A** of this assessment will be used in all psychomotor testing evaluations.

Students will be rated on **three items** and will receive a score for each item:

Yes, without prompting: **2 points**

Yes, with prompting: **1 point**

No: **0 points**

The possible range of scores for this assessment tool are 0 – 6, with 0 indicating the student demonstrated poor communication (even with prompting). A score of 4- 6 will indicate that the student demonstrated strong communication whereas a score of 1-3 will indicate that the student demonstrated poorer communications skills.

**Part B** of this assessment will be used in addition to Part A in psychomotor evaluation involves a real person.

Students will be rated on **four items** and will receive a score for each item:

Yes, without prompting: **2 points**

Yes, with prompting: **1 point**

No: **0 points**

The possible range of scores for this assessment tool are 0 – 8, with 0 indicating the student demonstrated poor communication (even with prompting). A score of 5-8 will indicate that the student demonstrated strong communication whereas a score of 1-4 will indicate that the student demonstrated poorer communications skills.
