# Supplementary material for: Evaluation of Incremental Validity of Casper in Predicting Program and National Licensure Performance of Undergraduate Nursing Students: Protocol for a Mixed Methods Study
Source: JMIR Res Protoc. 2023 Oct 18;12:e48672. doi: 10.2196/48672 (PMC10620628; doi:10.2196/48672)
Supplement: Multimedia Appendix 2 [file resprot_v12i1e48672_app2.docx]

**Assessment Tool: Problem Solving**

A problem was encountered?

No: do not complete assessment tool ______

Yes: complete assessment tool ____

| **Item** | **Yes, without prompting (2 points)** | **Yes, with prompting**  **(1 point)** | **No**  **(0 points)** | **Comments** |
| --- | --- | --- | --- | --- |
| 1. **The student recognized the problem** |  |  |  |  |
| 1. **The student attempted to address the problem** |  |  |  |  |
| 1. **The actions taken fixed the problem** |  |  |  |  |

**Directions and Scoring:**

This assessment will be completed by faculty evaluating students during psychomotor skills testing. Faculty will be provided with education regarding how to use this assessment tool.

If the student does not encounter a problem this tool will not be completed.

Students will be rated on **three items** and will receive a score for each item:

Yes, without prompting: **2 points**

Yes, with prompting: **1 point**

No: **0 points**

The possible range of scores for this assessment tool are 0 – 6, with 0 indicating the student did not recognize the problem (even with prompting). A score of 4- 6 will indicate that the student demonstrated strong problem solving skills whereas a score of 1-3 will indicate that the student demonstrated poorer problem solving.
